# Autoradiographic assessment of SynVesT-1 revealed changes in non-displaceable binding with demyelination and remyelination: implications for SV2A PET analysis

**DOI:** 10.1186/s13550-026-01391-2

**Published:** 2026-04-17

**Authors:** Pantila Panichnantakul, Robert C. Shaw, Valeria K. Burianova, Holly McErlain, Andrew Sutherland, Anna C. Williams, Adam D. Waldman, Adriana A.S. Tavares

**Affiliations:** 1 Centre for Clinical Brain Sciences, University of Edinburgh, Edinburgh, UK; 2 MS Society Edinburgh Centre of Excellence for MS Research, University of Edinburgh, Edinburgh, UK; 3 Centre for Cardiovascular Science, BHF-University of Edinburgh, University of Edinburgh, Edinburgh, UK; 4 School of Chemistry, University of Glasgow, Glasgow, UK; 5 Centre for Regenerative Medicine, Institute for Regeneration and Repair, University of Edinburgh, Edinburgh, UK; 6 Edinburgh Imaging, University of Edinburgh, Edinburgh, UK

## Introduction

Changes in synaptic density and function have been observed in a range of neurological disorders and are important determinants of disease progression and severity [1]. Synaptic vesicle glycoprotein 2 A (SV2A) is involved in the regulation of neurotransmitter release and is expressed in both excitatory and inhibitory synapse subtypes throughout the brain, making it a promising biomarker for assessing synaptic integrity [2, 3]. The use of positron emission tomography (PET) radioligands targeting SV2A has enabled in vivo visualisation of synaptic alterations [4]. In particular, [^18^F]SynVestT-1 has displayed excellent imaging and binding characteristics across various species, including mice, rats, non-human primates, and humans [5–9]. Although it has also been tested in a number of neurodegenerative and neuropsychiatric disorders [10, 11], variations in its binding dynamics under disease conditions and how those may contribute to reported outcome measures remain insufficiently characterised.

The cuprizone model is an established mouse model of global brain demyelination and remyelination. Loss of myelin occurs after 6 weeks of cuprizone administration, with significant remyelination occurring after discontinuation. While commonly used as a multiple sclerosis model, it is also characterised by pathology observed in many neurodegenerative diseases: myelin loss, axonal loss, glial cell-mediated inflammation, and alterations in synaptic density and plasticity [12, 13].

In this study, we tested [^3^H]SynVesT-1 in vitro using autoradiography due to its longer half-life and higher spatial resolution compared with [^18^F]SynVestT-1. We aimed to characterise its binding properties and how they are altered in demyelination and subsequent remyelination in this model. Understanding the extent to which pathological changes impact tracer behaviour will have important implications for interpreting in vivo PET imaging results using [^18^F]SynVesT-1.

## Materials and methods

### [^3^H]SynVesT-1 preparation

A seven-step synthesis of the [^3^H]SynVesT-1 precursor was developed and radiolabelled (NC064-87-3, Novandi Chemistry AB, Sweden) generating [^3^H]SynVesT-1 with a molar activity of 962 GBq/mmol (see supplementary materials).

### Animal care

All experiments conformed with the UK Home Office Animals Scientific Procedures Act 1986 with local ethical and veterinary approval (Bioresearch and Veterinary Services, University of Edinburgh). 12-week-old wildtype male C57BL/6J mice were obtained from Charles River and housed five mice per cage in temperature and humidity-controlled rooms on a 12 h light/dark cycle with *ad libitum* access to food and water.

### Cuprizone model

Fourteen adult male mice (12-week-old, weight 27.16 g±2.444 g) were acclimatised to a standard powdered rodent diet for one week before the addition of 0.2% cuprizone (C9012-25G, Sigma-Aldrich). After 6 weeks of cuprizone treatment (demyelination), five of the mice were culled for tissue and the remaining animals were returned to a normal pellet diet for an additional 6 weeks (five mice, early remyelination) or 18 weeks (four mice, late remyelination). Five age and sex-matched wildtype mice (weight 28.06 g±1.602 g) on a normal pellet diet were used as controls.

### [^3^H]SynVesT-1 autoradiography

All experiments were performed on fresh-frozen brains coronally sectioned at 10 μm thickness. Sections were incubated with the following concentrations of [^3^H]SynVesT-1: 100nM, 50nM, 20nM, 10nM, 5nM, 2.5nM, 1.25nM, and 0.5nM. Adjacent sections were co-incubated with 1µM of the non-radioactive ligand to determine non-specific binding (see supplementary materials).

### Image analysis

Autoradiography results were analysed using Fiji (ImageJ, NIH, USA). The optical density was measured in manually defined regions of interest (ROIs) using the Allen Brain Atlas as a guide. The whole slice and grey matter regions known to be affected by cuprizone treatment [12] were used as ROIs: cortex, caudate/putamen, hypothalamus. Duplicate samples were averaged. A calibration standard exposed alongside the samples was used to generate a standard curve to convert optical density measurements to µCi/g. These were then converted to the standard unit of fmol/mg using the molar activity of the ligand determined on the experimental day. Specific binding was estimated by subtracting the non-specific binding measurements from the total binding and curves were generated using a one site nonlinear regression for total and specific binding, and a simple linear regression for non-specific binding. From these, the receptor density (*B*_*max*_) and ligand affinity (*K*_d_) were determined. Lassen plots [14] were generated by plotting the specific binding against the total binding values for each ROI and fitted using a simple linear regression. The volume of non-displaceable binding (*V*_ND_) and percent occupancy were then determined from the x-intercept and slope of the graph, respectively. One mouse from each of the demyelination and early remyelination groups had a negative *V*_ND_ value and were thus excluded. *B*_*max*_ was converted from fmol/mg to nM assuming a brain density of 0.99 g/mL [15] and used to estimate the binding potential (BP) as the ratio between *B*_*max*_ and *K*_d_. Non-displaceable binding potential (*BP*_ND_) was calculated by subtracting the measured *V*_ND_ from the total *B*_*max*_ and normalising to *V*_ND_ [16].

### Statistical analyses

Statistical analyses were performed on Prism 10.2 (GraphPad, USA). All data was tested for normality using the Shapiro-Wilk test, and statistical outliers were identified and removed with Grubbs’ test. As all data were normally distributed, changes in *B*_*max*_, *K*_d_, BP, *V*_ND_, and *BP*_ND_ between conditions were analysed with one-way analysis of variance (ANOVA) and followed by Tukey’s multiple comparisons test when there was a significant effect. Data is shown as mean ± SEM and differences were considered significant if *p < 0.05.

## Results

**Fig. 1:**
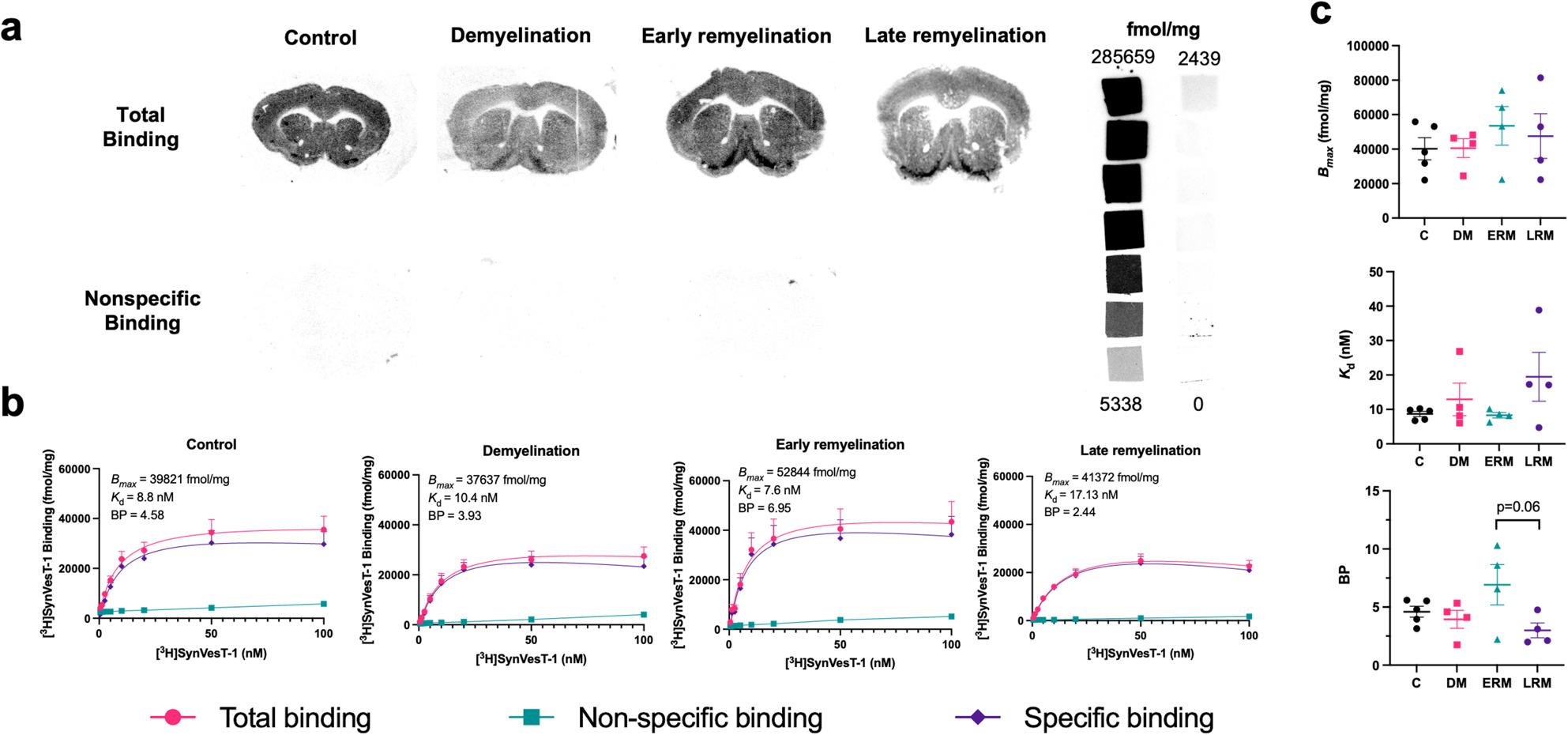
[^3^H]SynVesT-1 shows high sensitivity for its target under all conditions. (**a**) Representative autoradiograms of mouse brain sections incubated with 10 nM [^3^H]SynVesT-1 (top) and adjacent sections co-incubated with 1 µM of unlabelled SynVesT-1 (bottom) to determine the amount of non-specific binding. (**b**) Binding curves illustrating total, specific, and non-specific binding used to determine the *B*_*max*_, *K*_d_, and BP in each experimental group. (**c**) Comparison of *B*_*max*_ (top), *K*_d_ (middle), and BP (bottom) changes between groups. Data shown as mean ± SEM. Control (C) group *n* = 5, demyelination (DM) group *n* = 4, early remyelination (ERM) group *n* = 4, late remyelination (LRM) group *n* = 4

We found that the total binding saturated at concentrations above 20 nM and observed low non-specific binding values relative to total values when sections were co-incubated with the unlabelled ligand. Furthermore, we measured high levels of receptor density in control mice that tended to increase with early remyelination and recover with late remyelination. Binding potential measures likewise did not differ with demyelination, but tended to increase with early remyelination and then decrease at late remyelination (*p* = 0.06). We also observed consistently high ligand affinity in the nanomolar range during demyelination and early remyelination compared with controls, but noted an insignificant increase with late remyelination (Fig. 1).

**Fig. 2:**
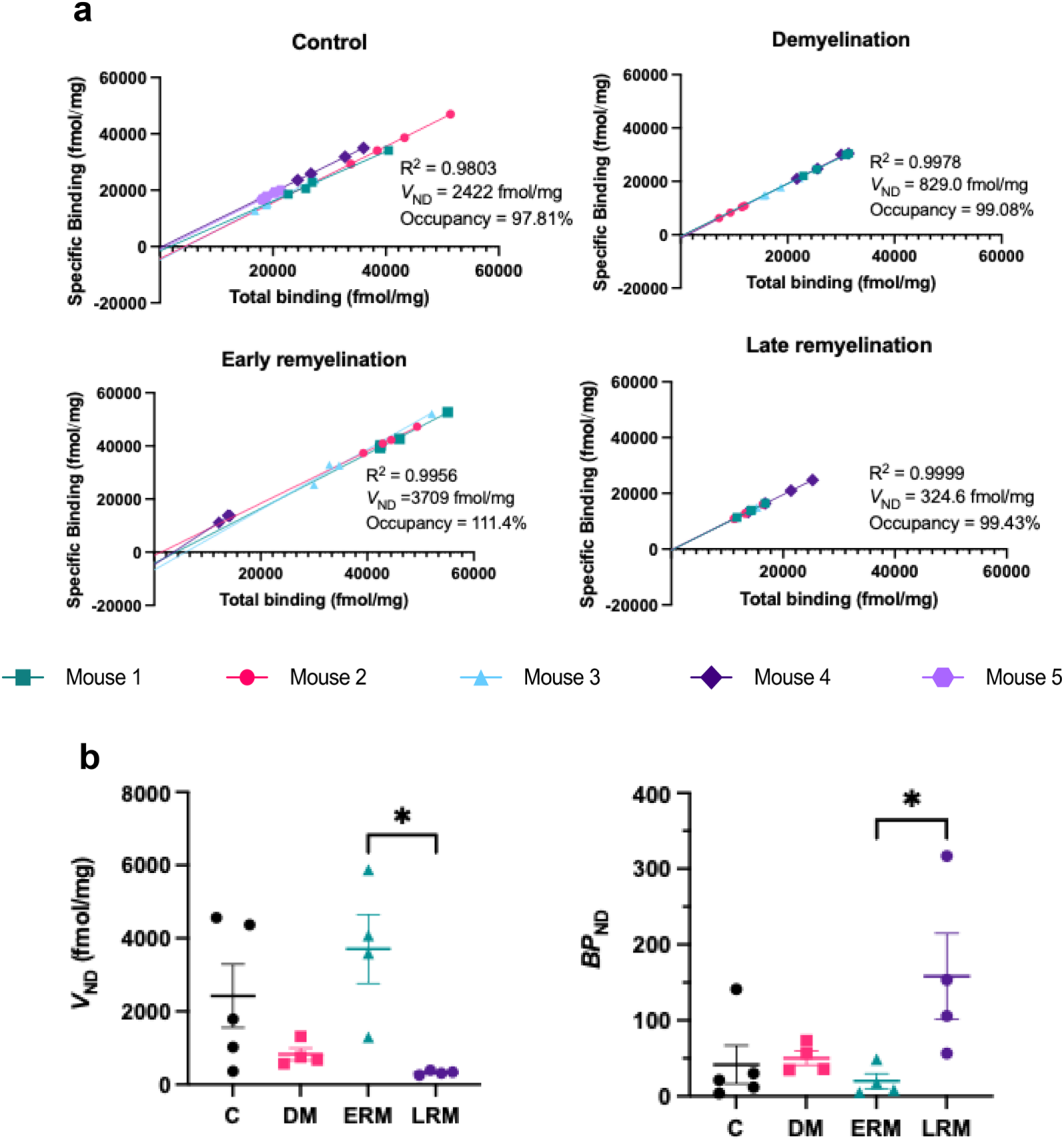
***V***_***ND***_ changes with demyelination and remyelination. (**a**) Lassen plots from sections incubated with 10 nM [^3^H]SynVesT-1. The non-displaceable volume of distribution (*V*_ND_) and receptor occupancy are represented by the x-intercept and slope of the regression line, respectively. Each point corresponds to a distinct region of interest from a single mouse (4 regions total). Reported values represent the mean of all mice within the respective experimental group. (**b**) Comparison of *V*_ND_ (left) and *BP*_ND_ (right) changes between groups. Data shown as mean ± SEM. One-way ANOVA followed by Tukey’s multiple comparisons, *p < 0.05. Control (C) group *n* = 5, demyelination (DM) group *n* = 4, early remyelination (ERM) group *n* = 4, late remyelination (LRM) group *n* = 4

Lassen plots generated from the total and specific binding values obtained from each ROI at the measured *K*_d_ (10nM) revealed very low *V*_ND_ values, representing less than 10% of total receptor binding under all conditions. *V*_ND_ tended to decrease with demyelination and late remyelination, but increase with early remyelination compared with controls, and was significantly decreased with late remyelination compared with early remyelination (*p* = 0.02). High receptor occupancy was measured at all timepoints and *V*_ND_-corrected *BP*_ND_ was significantly increased with late remyelination compared with early remyelination (*p* = 0.04) (Fig. 2).

## Discussion

Autoradiography on brain tissue from mice treated with cuprizone showed that the binding properties of [^3^H]SynVesT-1 are altered in states of demyelination and remyelination. Our results demonstrate very low non-specific binding coupled with high binding potential and ligand affinity comparable to previous reports in healthy control mice with [^18^F]SynVesT-1 PET and autoradiography [7, 17]. Although not statistically significant, we noted a trend toward higher *K*_d_ values with late remyelination relative to other timepoints. This may reflect slight changes in protein conformation under these conditions that can affect tracer affinity for SV2A, though further work is required to confirm this. Moreover, we observed non-significant variations in *B*_*max*_, notably slight increases with early remyelination and recovery with late remyelination, along with trending decreases in BP and significantly higher *BP*_ND_ at late remyelination compared with early remyelination. While higher *K*_d_ could have impacted BP measurements at this timepoint, these binding changes may be reflecting changes in synaptic density and activity that are known to occur in the cuprizone model [18, 19]. Surprisingly, no significant change with demyelination was observed in our measures, though no study to our knowledge has examined SV2A synapses specifically in this model. Future work involving histological assessments of SV2A expression should thus be done to provide further insight into these observations. Overall, however, these results confirm that this tracer is highly sensitive for its target, highlighting its potential as an in vivo biomarker of synaptic pathology.

We found that *V*_ND_ was highly variable between conditions; values were lower in the demyelination and late remyelination groups, and higher in the early remyelination group compared with controls. Notably, *V*_ND_ is typically assumed to be uniform across brain regions and pathological conditions in vivo. Deviations from these assumptions can therefore introduce analysis biases since [^18^F]SynVesT-1 PET binding is commonly described by fitting the simplified reference tissue model with non-displaceable binding potential (*BP*_ND_ = (*V*_T_-*V*_ND_)/*V*_ND_) as the primary outcome measure, or with the total distribution volume (*V*_T_ = *V*_ND_ + *V*_S_) derived from the one and two-tissue compartment models [6, 7, 16, 17, 20]. These variations in *V*_ND_ can thus lead to over- or underestimation of tracer binding and misinterpretation of outcomes, as demonstrated by our *V*_ND_-corrected *BP*_ND_ estimates, which showed significantly different trends compared with standard BP measures. These findings thus highlight the necessity for further investigations into the relationship between *V*_ND_ and specific pathological states, and the need for appropriate kinetic modelling corrections to ensure reliable quantification of outcomes.

The apparent changes in *V*_ND_ may be linked to significant tissue alterations that are known to occur with cuprizone treatment, with not only changes in brain myelin, but also glial activation, and alterations in axonal and synaptic density during both de- and remyelination [12, 21]. These neuroinflammatory and neurodegenerative changes may impact the availability of non-specific binding sites in each condition and overall affect the distribution and binding potential of [^3^H]SynVesT-1, resulting in varying levels of *V*_ND_.

A limitation of this study is the small sample size used, as this can increase the margin of error and reduce the statistical power of our analyses. Although we did not find statistically significant differences between timepoints for many of our metrics due to variability between samples, we observed clear trends that are generally in line with what is known about both the tracer and pathology in the cuprizone model. Another notable limitation of our study is that Lassen plot estimations of *V*_ND_ assume uniformity across brain regions [22], which has previously been shown to be an inaccurate assumption [23, 24]. Although we did not observe deviations from linearity in our ROIs that would suggest regional differences, additional studies are needed to characterise more precisely the significance of spatial variations in *V*_ND_ with this tracer. The *V*_ND_ values measured in this study may also underestimate in vivo levels due to routine washing steps that may reduce nonspecifically bound and free radioligand in tissue. Nevertheless, these results demonstrate that significant changes in *V*_ND_ can occur with tissue pathology, challenging the assumption that *V*_ND_ is uniform across varying conditions.

## Conclusion

Variations in *V*_ND_ with pathology may be a confounding factor in imaging measurements with [^18^F]SynVestT-1. These variations may affect quantification of true changes in receptor binding between experimental groups. There is thus a need for further investigations into *V*_ND_ changes in other disease states and SV2A tracers to validate imaging findings.

## Supplementary Information

Below is the link to the electronic supplementary material.

Supplementary Material 1

## Data Availability

The datasets generated and/or analysed during the current study are available from the corresponding author on reasonable request.

## Abbreviations

ANOVA: Analysis of variance
B_max_: Receptor density
BP: Binding potential
BP_ND_: Non-displaceable binding potential
C: Control
DM: Demyelination
ERM: Early remyelination
K_d_: Ligand affinity
LRM: Late remyelination
PET: Positron emission tomography
RM: Remyelination
ROI: Region of interest
SEM: Standard error of the mean
SV2A: Synaptic vesicle glycoprotein 2 A
V_ND_: Non-displaceable binding volume
V_S_: Specifically bound volume of distribution
V_T_: Total volume of distribution
