# Supplementary material for: Autoradiographic assessment of SynVesT-1 revealed changes in non-displaceable binding with demyelination and remyelination: implications for SV2A PET analysis
Source: EJNMMI Res. 2026 Apr 17;16:90. doi: 10.1186/s13550-026-01391-2 (PMC13234051; doi:10.1186/s13550-026-01391-2)
Supplement: Supplementary file 1 — Supplementary Material 1 [file 13550_2026_1391_MOESM1_ESM.docx]

**Supplementary material**

**[^3^H]SynVesT-1 Preparation**

**Synthesis of Precursor and [^3^H]SynVesT-1.**

**General Synthetic Experimental Section**

All reagents and starting materials were obtained from commercial sources and used as received unless otherwise stated. Dry solvents were purified using a PureSolv 500 MD solvent purification system. All reactions were performed under an atmosphere of air unless otherwise stated. Dry glassware was oven-dried at 140 °C for a minimum of 16 h, cooled to room temperature *in vacuo* and then purged with argon. Brine is defined as a saturated aqueous solution of sodium chloride. Merck aluminium-backed plates pre-coated with silica gel 60 (UV_254_) were used for thin layer chromatography and were visualized under UV light (254/365 nm) then stained with iodine, potassium permanganate, vanillin or ninhydrin solution. Flash column chromatography was carried out using Merck Geduran Si 60 (40‒63 µm). ^1^H and ^13^C NMR spectra were recorded on a 400 MHz spectrometer with chemical shift values reported in ppm relative to tetramethylsilane (*δ*_H_ 0.00 and *δ*_C_ 0.0) or CHCl_3_ (*δ*_H_ 7.26 and *δ*_C_ 77.2). Assignments of ^13^C NMR signals are based on DEPT experiments. Mass spectra were obtained using electrospray (ESI) techniques. Melting points were determined on a Gallenkamp melting point apparatus and are uncorrected. Infrared spectra were recorded neat on a Shimadzu FTIR-84005 spectrometer. Optical rotations were determined as solutions irradiating with the sodium D line (λ = 598 nm) using an Autopol V polarimeter. [α]_D_ values are reported in units 10^−1^ deg cm^2^ g^−1^. Chiral HPLC methods were calibrated with the corresponding racemic mixtures.

(*E*)-3-(3',5'-Difluoro-4'-iodophenyl)prop-2-enal

In the oven-dried flask under argon, *N*,*N*-diisopropylethylamine (1.80 mL, 12.8 mmol) was dissolved in anhydrous tetrahydrofuran (18 mL). The resultant solution was cooled to −10 °C and a solution of *n*-butyllithium (4.90 mL, 2.5 M in hexane, 12.3 mmol) was added dropwise. The mixture was stirred for 0.5 h at −10 °C. The resulting solution was added dropwise to a solution of 3,5-difluorobenzonitrile (1.03 g, 7.40 mmol) in anhydrous tetrahydrofuran (12 mL) at −78 °C under argon. After 10 minutes, a solution of iodine (2.25 g, 8.87 mmol) in anhydrous tetrahydrofuran (6 mL) was added dropwise. The reaction mixture was slowly warmed to room temperature and left stirring overnight under argon. After 18 h, the mixture was treated with a 10% aqueous solution of sodium thiosulfate (15 mL) and extracted with hexane/ethyl acetate mixture (v/v, 1:1, 3 × 24 mL). The combined organic layers were dried (MgSO_4_), filtered, concentrated and dried *in vacuo*. The resulting 3,5-difluoro-4-iodobenzonitrile was obtained as a brown oil and was used without further purification. Diisobutylaluminium anhydride (9.10 mL, 1 M in dichloromethane, 9.10 mmol) was then added dropwise to a solution of 3,5-difluoro-4-iodobenzonitrile (2.02 g, 7.62 mmol) in anhydrous dichloromethane (22 mL) under argon at 0 °C. The reaction was slowly warmed to room temperature, stirred for 2 h, and then quenched with an aqueous solution of 6 M hydrochloric acid (20 mL). The mixture was extracted with dichloromethane (3 × 50 mL). The combined organic layers were passed through Celite^®^, dried (MgSO_4_), filtered, concentrated, and dried *in vacuo* to afford 3,5-difluoro-4-iodobenzaldehyde as a white solid, which was used without further purification. 3,5-Difluoro-4-iodobenzaldehyde (1.50 g, 5.63 mmol) and (triphenylphosphoranylidene)acetaldehyde (1.88 g, 6.19 mmol) were dissolved in anhydrous tetrahydrofuran (60 mL) under argon. The reaction mixture was heated to 50 °C and stirred for 18 h. The crude mixture was cooled to room temperature and concentrated *in vacuo*. The resulting residue was dissolved in ethyl acetate (40 mL) and washed with water (20 mL). The aqueous layer was extracted with ethyl acetate (2 × 40 mL). The combined organic layers were dried (MgSO_4_), filtered and concentrated *in vacuo*. Purification by flash column chromatography eluting with 10% diethyl ether in petroleum ether (40‒60) gave (*E*)-3-(3',5'-difluoro-4'-iodophenyl)prop-2-enal as a pale-yellow solid (1.16 g, 53% over 3 steps). Mp 152−154 °C; IR (neat) 2923, 2356, 1663, 1559, 1422, 1128, 1020 cm^−1^; ^1^H NMR (400 MHz, CDCl_3_) *δ* 6.72 (1H, dd, *J* = 16.0, 7.5 Hz, 2-H), 7.06−7.11 (2H, m, 2'-H and 6'-H), 7.36 (1H, d, *J* = 16.0 Hz, 3-H), 9.74 (1H, d, *J* = 7.5 Hz, 1-H); ^13^C{^1^H} NMR (101 MHz, CDCl_3_) *δ* 74.3 (t, ^2^*J*_CF_ = 29.7 Hz, C), 110.7 (dd, ^2^*J*_CF_ = 25.9 Hz, ^4^*J*_CF_ = 2.6 Hz, 2 × CH), 130.7 (CH), 137.0 (t, ^3^*J*_CF_ = 9.2 Hz, C), 148.5 (t, ^4^*J*_CF_ = 3.0 Hz, CH), 163.4 (dd, ^1^*J*_CF_ = 248.3 Hz, ^3^*J*_CF_ = 6.3 Hz, 2 × C), 192.7 (CH); MS (ESI) *m*/*z* 293 (M‒H^‒^, 100); HRMS (ESI) *m/z*: [M ‒ H]^‒^ Calcd for C_9_H_4_F_2_IO 292.9280; Found 292.9277.

Methyl (3*R*)-3-(3',5'-difluoro-4'-iodophenyl)-4-nitrobutanoate

To a stirred solution of (*E*)*-*3-(3',5'-difluoro-4'-iodophenyl)prop-2-enal (0.18 g, 0.61 mmol) in methanol (1.3 mL) was added (*R*)-α,α-bisphenyl-2-pyrrolidinemethanol trimethylsilyl ether (0.020 g, 0.061 mmol), benzoic acid (0.015 g, 0.12 mmol) and nitromethane (0.21 mL, 1.8 mmol) at room temperature. The reaction mixture was stirred for 21 h and then cooled to 0 °C. *N*-Bromosuccinimide (0.14 g, 0.80 mmol) was added, and the mixture was stirred for another 18 h slowly warming up to 15 ºC. The mixture was then concentrated *in vacuo*. Purification by flash column chromatography eluting with 60% dichloromethane in hexane gave methyl (3*R*)-3-(3',5'-difluorophenyl)-4-nitrobutanoate as a colourless oil, which solidified upon standing (0.094 g, 40% over two steps). IR (neat) 2958, 2360, 1732, 1554, 1431, 1373, 1203, 1018, 856 cm^−1^; [α]_D_^26^ +1.3 (*c* 0.1, CHCl_3_); ^1^H NMR (400 MHz, CDCl_3_) *δ* 2.72 (1H, dd, *J* = 16.8, 7.6 Hz, 2-*H*H), 2.77 (1H, dd, *J* = 16.8, 7.2 Hz, 2-H*H*), 3.67 (3H, s, OCH_3_), 3.93‒4.03 (1H, m, 3-H), 4.62 (1H, dd, *J* = 13.0, 8.3 Hz, 4-*H*H), 4.73 (1H, dd, *J* = 13.0, 6.5 Hz, 4-H*H*), 6.81 (1H, d, *J* = 6.1 Hz, 2'-H and 6'-H); ^13^C{^1^H} NMR (101 MHz, CDCl_3_) *δ* 36.9 (CH_2_), 39.4 (t, ^4^*J*_CF_ = 2.0 Hz, CH), 52.3 (CH_3_), 70.6 (t, ^2^*J*_CF_ = 29.4 Hz, C), 78.4 (CH_2_), 110.7 (dd, ^2^*J*_CF_ = 25.6 Hz, ^4^*J*_CF_ = 2.7 Hz, 2 × CH), 142.2 (t, ^3^*J*_CF_ = 8.5 Hz, C), 163.0 (dd, ^1^*J*_CF_ = 248.5 Hz, ^3^*J*_CF_ = 6.3 Hz, 2 × C), 170.4 (C); MS (ESI) *m*/*z* 366 (M − [H_2_O] − H^−^, 100); HRMS (ESI) *m/z*: [M − [H_2_O] − H]^−^ Calcd for C_11_H_7_F_2_INO_3_ 365.9444; Found 365.9438. The enantiomeric ratio was determined by HPLC analysis with a CHIRALCEL^®^ AD-H column (96:4, hexane:*i*PrOH, flow rate of 1.5 mL/min): t_minor_ = 9.68 min, t_major_ = 10.73 min; 97:3 er.

(4*R*)-4-(3',5'-Difluoro-4'-iodophenyl)pyrrolidin-2-one

To a stirred suspension of methyl (3*R*)-3-(3',5'-difluoro-4'-iodophenyl)-4-nitrobutanoate (0.127 g, 0.490 mmol) in a mixture of ethanol and water (4.5 mL, 2:1, v/v) was added ammonium chloride (0.785 g, 14.7 mmol) and iron powder (0.277 g, 4.90 mmol). After stirring for 3 h at room temperature, the reaction mixture was adjusted to pH 14 with an aqueous solution of 6 M sodium hydroxide (2 mL) and extracted with ethyl acetate (3 × 50 mL). The combined organic layers were dried over MgSO_4_ and concentrated *in vacuo*. Purification by flash column chromatography eluting with 5% methanol in diethyl ether gave (4*R*)-4-(3',5'-difluoro-4'-iodophenyl)pyrrolidin-2-one as a white solid (0.061 g, 63%). IR (neat) 2360, 1743, 1689, 1431, 1365, 1203, 1011, 852 cm^−1^; [α]_D_^26^ −10.9 (*c* 0.1, CHCl_3_); ^1^H NMR (400 MHz, CDCl_3_) *δ* 2.43 (1H, dd, *J* = 16.9, 8.2 Hz, 3-*H*H), 2.76 (1H, dd, *J* = 16.9, 9.0 Hz, 3-H*H*), 3.39 (1H, dd, *J* = 9.6, 6.7 Hz, 5-*H*H), 3.63‒3.73 (1H, m, 4-H), 3.76‒3.84 (1H, m, 5-H*H*), 6.47 (1H, br s, NH), 6.82 (2H, d, *J* = 7.0 Hz, 2'-H and 6'-H); ^13^C{^1^H} NMR (101 MHz, CDCl_3_) *δ* 37.4 (CH_2_), 39.7 (t, ^4^*J*_CF_ = 2.2 Hz, CH), 48.8 (CH_2_), 69.0 (t, ^2^*J*_CF_ = 29.6 Hz, C), 110.0 (dd, ^2^*J*_CF_ = 25.3 Hz, ^4^*J*_CF_ = 2.7 Hz, 2 × CH), 146.2 (t, ^3^*J*_CF_ = 8.5 Hz, C), 163.0 (dd, ^1^*J*_CF_ = 247.8 Hz, ^3^*J*_CF_ = 6.5 Hz, 2 × C), 176.6 (C); MS (ESI) *m*/*z* 323 (M + H^+^, 100). HRMS (ESI) *m/z*: [M + H]^+^ Calcd for C_10_H_9_F_2_INO 323.9691; Found 323.9694.

(4*R*)-4-(3',5'-Difluoro-4'-iodophenyl)-1-[(3"-methylpyridin-4"-yl)methyl]pyrrolidin-2-one

In an oven-dried flask under argon, sodium hydride (0.013 g, 0.37 mmol, 60% dispersion in mineral oil) was added, washed with hexane (1 mL) and dried at the room temperature *in vacuo* for 0.5 h. The sodium hydride was suspended in anhydrous tetrahydrofuran (0.1 mL) and cooled to 0 ºC. A solution of (4*R*)-4-(3',5'-difluoro-4'-iodophenyl)pyrrolidin-2-one (0.048 g, 0.15 mmol) in anhydrous tetrahydrofuran (1.3 mL) was added to the mixture, followed by 4-(chloromethyl)-3-methylpyridine hydrochloride (0.030 g, 0.16 mmol) and tetrabutylammonium iodide (0.0030 g, 0.0074 mmol). The reaction mixture was warmed to room temperature and stirred for 18 h. After cooling to 0 °C, the reaction was quenched with a saturated solution of sodium hydrogencarbonate (1.5 mL) and extracted with chloroform (3 × 5 mL). The combined organic layers were dried (MgSO_4_), filtered and concentrated *in vacuo*. Purification by flash column chromatography eluting with 7% methanol in diethyl ether gave (4*R*)-4-(3',5'-difluoro-4'-iodophenyl)-1-[(3"-methylpyridin-4"-yl)methyl]pyrrolidin-2-one as a colourless oil, which solidified upon standing (0.015 g, 23%). IR (neat) 2360, 1743, 1689, 1574, 1427, 1362, 1203, 1011, 852 cm^−1^; [α]_D_^26^ +37.2 (*c* 0.1, CHCl_3_); ^1^H NMR (400 MHz, CDCl_3_) *δ* 2.30 (3H, s, 3''-CH_3_), 2.58 (1H, dd, *J* = 17.0, 8.0 Hz, 3-*H*H), 2.93 (1H, dd, *J* = 17.0, 8.8 Hz, 3-H*H*), 3.19–3.27 (1H, m, 5-*H*H), 3.53‒3.68 (2H, m, 4-H and 5-H*H*), 4.41 (1H, d, *J* = 15.4 Hz, 7''-*H*H), 4.63 (1H, d, *J* = 15.4 Hz, 7''-H*H*), 6.74 (2H, d, *J* = 6.6 Hz, 2'-H and 6'-H), 7.04 (1H, d, *J* = 5.0 Hz, 5"-H), 8.41‒8.45 (2H, m, 2"-H and 6"-H); ^13^C{^1^H} NMR (101 MHz, CDCl_3_) *δ* 16.0 (CH_3_), 36.8 (t, ^4^*J*_CF_ = 2.0 Hz, CH), 38.0 (CH_2_), 43.6 (CH_2_), 53.2 (CH_2_), 69.2 (t, ^2^*J*_CF_ = 29.5 Hz, C), 109.9 (dd, ^2^*J*_CF_ = 25.3 Hz, ^4^*J*_CF_ = 2.6 Hz, 2 × CH), 122.4 (CH), 131.6 (C), 142.6 (C), 145.8 (t, ^3^*J*_CF_ = 8.4 Hz, C), 148.1 (CH), 151.4 (CH), 163.0 (dd, ^1^*J*_CF_ = 248.2 Hz, ^3^*J*_CF_ = 6.5 Hz, 2 × C), 172.9 (C); MS (ESI) *m*/*z* 429 (M + H^+^, 100); HRMS (ESI) *m/z*: [M + H]^+^ Calcd for C_17_H_16_F_2_IN_2_O 429.0270; Found 429.0269.

**Radioligand Preparation.** [^3^H]SynVesT-1 was custom synthesised by NOVANDI Chemistry AB (Sweden, Batch No. NC064-87-3) using (4*R*)-4-(3',5'-difluoro-4'-iodophenyl)-1-[(3"-methylpyridin-4"-yl)methyl]pyrrolidin-2-one prepared in our lab with a molar activity of 26 Ci/mmol (1.0 TBq/mmol). The radiosynthesis of [^3^H]SynVesT-1 was accomplished with a radiochemical purity of >99%, a stereochemical purity of >99% and a radioactive concentration of 1.0 mCi/mL (38 MBq/mL) in ethanol.

**Chiral and Racemic HPLC Traces**

**(*R*)-Enantiomer:**

**Racemate:**

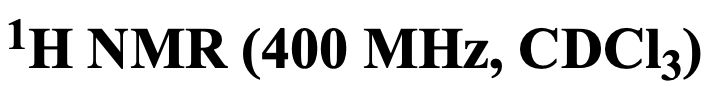
**^1^H and ^13^C NMR Spectra for all Compounds**

**
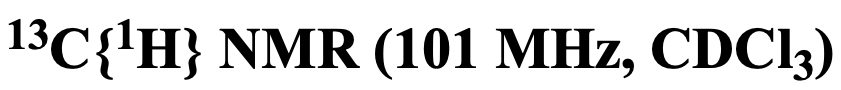
**

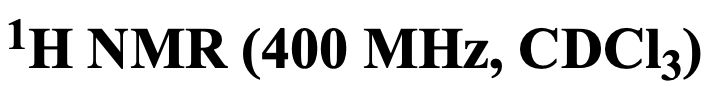

**
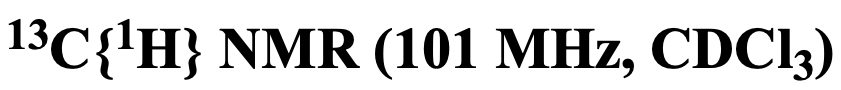
**

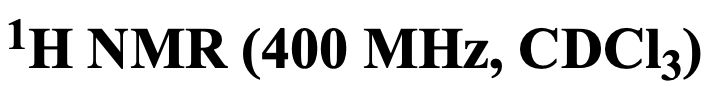

**
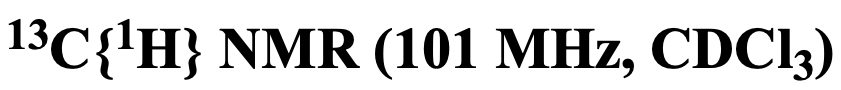
**

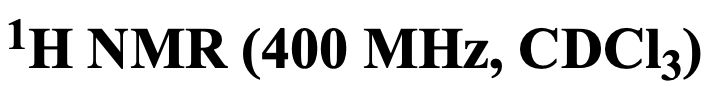


**
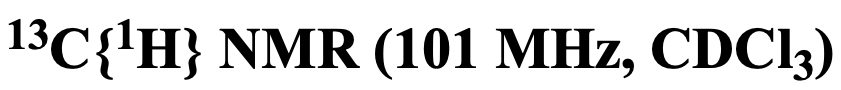
**

**Tissue collection and preparation**

At the desired timepoints, mice were given a lethal dose of pentobarbital sodium (Dolethal, 200 mg/mL, Vetoquinol UK Ltd) intraperitoneally before transcardial perfusion with phosphate-buffered saline (PBS). Brains were then removed, embedded in optimal cutting temperature medium (KMA-0100-00A, CellPath), and immediately frozen in 5-methylbutane chilled on dry ice. Samples were sectioned at a thickness of 10 μm on a Leica cryostat, mounted onto Superfrost^TM^ Plus slides (Thermo Fisher Scientific, Waltham, MA), and stored at -80 ˚C until further use.

**[^3^H]SynVesT-1 Autoradiography**

Brain sections were washed in ice-cold 1x PBS (3 x 5 mins) and incubated at room temperature for 1 hour using the following concentrations of [^3^H]SynVesT-1 (NC064-87-3, Novandi Chemistry AB, Sweden): 100 nM, 50 nM, 20 nM, 10 nM, 5 nM, 2.5 nM, 1.25 nM, and 0.5 nM prepared in 1x PBS and 1% ethanol. To determine the amount of non-specific binding, adjacent sections were incubated with the same range of concentrations, but with the addition of 1 µM of the non-radioactive ligand. Next, sections were washed with ice-cold 1x PBS (3 x 5 mins), briefly dipped in distilled water, and allowed to dry overnight. The following day, air-dried sections were exposed to a BAS Storage Phosphor Screen (20 x 40 cm, 28956481, Cytiva, USA) for 1 week before being scanned with a Typhoon Phosphor Trio imaging system (Typhoon FLA 7000, Biomolecular imager, Cytiva, USA).

**Supplementary Fig. 1** Binding curves of individual mice in each experimental group illustrating total, specific, and non-specific binding of [^3^H]SynVesT-1

**Supplementary Fig. 2** Lassen plots of individual mice in each experimental group. Total and specific binding values were obtained from sections incubated with 10 nM [^3^H]SynVesT-1. The non-displaceable volume of distribution (*V_ND_*) and receptor occupancy are represented by the x-intercept and slope of the regression line, respectively. Each point corresponds to a distinct region of interest (4 regions total)
